# Pre-operative administration of butorphanol mitigates emergence agitation in patients undergoing functional endoscopic sinus surgery: A randomized controlled clinical trial

**DOI:** 10.3389/fpsyt.2022.1090149

**Published:** 2023-01-17

**Authors:** Xiao Zhang, Siyi Qi, Zhen Lin, Yizhe Zhang, Wanbing Dai, Weitian Tian, Jie Tian, Li Zheng, Diansan Su, Xiaorong Huai

**Affiliations:** ^1^Department of Anesthesiology, Renji Hospital, School of Medicine, Shanghai Jiao Tong University, Shanghai, China; ^2^Department of Anesthesiology, Quanzhou First Hospital Affiliated to Fujian Medical University, Quanzhou, China

**Keywords:** trachea extubation complications, butorphanol, post-operative recovery, general anesthesia, emergence agitation

## Abstract

**Background:**

This study explored the effectiveness of pre-operative intravenous injection of butorphanol in the alleviation of emergence agitation (EA) in patients undergoing functional endoscopic sinus surgery (FESS).

**Methods:**

Patients (*n* = 708) were randomized into two groups. The butorphanol group (Group B, *n* = 358) received butorphanol infusion (20 ug/kg) before anesthesia induction, while the control group (Group C, *n* = 350) received an equal volume of normal saline infusion. General anesthesia was induced with sufentanil, propofol, and rocuronium, and was maintained with sevoflurane and remifentanil. Vasoactive drugs maintained the hemodynamic indices within 20% of the baseline.

**Results:**

The incidence of EA was significantly lower in Group B than that in Group C (Group B vs. C: 24.3% vs. 31.4%, respectively; *P* = 0.034). The times to spontaneous breathing (26.5 min vs. 23.7 min, *P* = 0.011), verbal response (36.0 min vs. 33.4 min, *P* = 0.012), and extubation (31.0 min vs. 28.7 min, *P* = 0.025) were longer in Group B, and the grade of cough (0.33 vs. 0.43, *P* = 0.024) at extubation in Group B was lower than that in Group C (*P* = 0.024). The mean arterial pressure at the end of the operation (*P* = 0.004) and at 5 min after extubation (*P* = 0.008) was higher and hypotension was less prominent (0.6% vs. 2.6%, *P* = 0.030) in Group B.

**Conclusion:**

Pre-operative intravenous injection of butorphanol decreased the incidence of EA after FESS and provided smooth and hemodynamically stable emergence without extending the stay in post-anesthesia care unit.

**Clinical trial registration:**

https://www.clinicaltrials.gov/, identifier NCT03398759.

## Background

Perioperative Neurocognitive Disorders (PND) encompass cognitive impairment existing pre-operatively, post-operative delirium, delayed neurocognitive disorder (dNCR), and post-operative neurocognitive disorder (NCD) (1). Research into cognitive change affecting patients after anesthesia and surgery has accelerated in recent years. Emergence agitation (EA), also known as emergence delirium, which is also recognized as PND, is a common symptom after ear, nose, and throat (ENT) surgery under general anesthesia, especially in children and patients over 65 years old, characterized by aimless restlessness, hallucination, delusion, inconsolable crying or moaning, disorientation, and incoherence (2–6). Although the exact mechanism of EA has not been clarified after decades of research, its incidence varies from 5 to 80% and can be affected by many factors, such as age, gender, types of operation, volatile anesthetics, benzodiazepine premedication, pre-operative anxiety, post-operative pain, and others (7–10). EA can increase the risk of injury, pain, hemorrhage, self-extubation, or removal of catheters. This can lead to serious complications, such as hypoxia, aspiration pneumonia, bleeding, or reoperation (2, 3, 9). Although many strategies had been proven helpful to the reduction of the incidence of EA, like α-2-adrenoreceptor agonists (11, 12), total intravenous anesthesia (13), and multimodal analgesia (14), these preventive strategies often yielded inconsistent results depending on the methodology of the study and the patients assessed (15), which led to residual sedation and hemodynamic changes that resulted in prolonged post-anesthesia care unit (PACU) stay.

Butorphanol is a mixed opioid agonist–antagonist, with strong κ-receptor agonist and weak μ-receptor antagonist activities (16). It is commonly used for the management of cancer, post-operative, gynecological, and obstetric pain. Additionally, butorphanol elicits less pronounced respiratory depression and sedation effects. This renders butorphanol as a good medication for the alleviation of agitation. However, there is no clinical evidence that confirms the effectiveness of butorphanol.

In this randomized, double-blind, placebo-controlled study, the hypothesis that pre-operative intravenous injection of butorphanol would reduce the incidence of EA in adult patients undergoing FESS was evaluated. Furthermore, we evaluated the effects of butorphanol on the quality of recovery after FESS.

## Methods

This single-center, prospective, randomized, double-blinded clinical trial was conducted at the Renji Hospital (affiliated to the Shanghai Jiao Tong University School of Medicine) from February 2018 to May 2020. This study was approved by Renji Hospital Ethics Committee (2017-159) and was registered at clinicaltrials.gov (NCT03398759). Written informed consent was obtained from all patients before inclusion.

### Participants

Patients aged 18–65 years and American Society of Anesthesiologists physical status class I–II, who were scheduled for FESS under general anesthesia were included in the study. The excluding criteria are: (1) body mass index (BMI) > 30 kg/m^2^; (2) cerebral disease or patients with a history of neurological and psychiatric diseases, including Alzheimer disease, stroke, epilepsy, and psychosis; (3) bradycardia (heart rate < 60 beats per minute for any reasons); (4) gastrointestinal ulcer; (5) urinary incontinence; (6) asthma or chronic obstructive pulmonary disease; (7) allergy to butorphanol; (8) auditory or vision disorders; (9) unwillingness to comply with the protocol or procedures; and (10) inability to communicate in Chinese Mandarin.

### Randomization and blinding

A biostatistician who did not participate in the data management and statistical analyses generated the random sequences. The PROC PLAN program (SAS, version 9.0) was used to generate the sample randomization sequence using 1:1 allocation with block 90 and a length = 8. The results of the randomization were sealed in sequentially numbered envelopes. Consecutively recruited patients were assigned to Groups B or C. Group B received an intravenous injection of butorphanol (trade name Nuoyang, produced by Jiangsu Hengrui Pharmaceutical Co., Ltd., Lianyungang, China) at a dose of 20 ug/kg (diluted with normal saline to 1 mg/ml) before anesthesia induction, while Group C received an equal volume of normal saline infusion as the placebo at the same time point. The investigator, attending anesthetist, surgeons, recovery, ward nurses, and patients were blinded to group assignment.

### Sample size estimation

The sample size was calculated based on the estimated differences of EA incidence between the two groups with PASS (ver. 11.0) (two independent proportions, *z*-test). With an alpha = 0.05, power = 0.8, an expected reduction in the EA incidence from 35 to 25% (17). With degree-of-freedom = 1 and attrition rate = 10%, we estimated that 358 patients were needed in each group.

### Study design

After the patients arrived at the OR, routine monitors, including the electrocardiogram, pulse oxygen saturation (SpO_2_), non-invasive arterial pressure were applied upon patient arrival in the operating room. The room temperature was kept at 20°C–24°C during the operation. One min before the induction of anesthesia, the patient was administrated with a drug labeled “experimental drug” according to the patient’s weight (20 ug/kg of butorphanol or the same volume of normal saline). General anesthesia was induced by the combined use of sufentanil (0.5 ug/kg), propofol (2 mg/kg), and rocuronium (0.6 mg/kg). The orotracheal intubation was then performed, the tidal volume of mechanical ventilation was set to 6–8 ml/kg, and the ventilation frequency was adjusted to 12–15 times/min to maintain ETCO_2_ between 35 and 40 mmHg (1 mmHg = 0.133 kPa). Anesthesia was maintained initially with sevoflurane based on 1.3 age-adjusted minimum alveolar concentration combined with remifentanil (0.2–0.5 ug/kg/min), and sevoflurane and remifentanil were adjusted to maintain the bispectral index (BIS) (A-2000TM SP, Aspect Medical Systems, Norwood, MA, USA) values between 40 and 60. We would decrease the dose of sevoflurane and remifentanil when hypotension happened. Vasoactive drugs were also used to maintain the hemodynamic indices of patients within 20% of the baseline. Ephedrine was first considered to increase blood pressure. If hypotension was not revised, then, phenylephrine was chosen to increase blood pressure. If the above vasoactive drugs had no effect on increase blood pressure, norepinephrine, or epinephrine was used to raise blood pressure.

Anesthetic maintenance drugs continued until leaving the operating room. All patients were transferred to PACU for extubation.

After entering the PACU, the arterial pressure, electrocardiogram and SpO_2_ were continuously monitored, and the patients were mechanically ventilated. All patients were given atropine (15 ug/kg) and neostigmine (50 ug/kg) to antagonize the residual muscle relaxation. Extubation was performed when patients began breathing spontaneously and were able to sensitively and accurately respond to commands of nurses or doctors. All data were recorded in the CRF table.

Patients were discharged from the PACU when their Aldrete score ≥ 9 (18).

Emergence duration is defined as the time spent in the PACU. During emergence, the level of agitation was evaluated by a nurse using the Ricker sedation–agitation scale (SAS). The agitation score of patient was recorded based on the following: 1 = minimal or no response to noxious stimuli, 2 = arousal to physical stimuli but no communication, 3 = difficult to arouse but awaken to verbal stimuli or gentle shaking, 4 = calm and follows commands, 5 = anxious or physically agitated and calm to verbal instructions, 6 = requiring restraint and frequent verbal reminding of limits, and 7 = pulling at tracheal tube and trying to remove catheters or striking staff members (19). EA was defined as the highest SAS score ≥ 5 during emergence, and SAS score > 5 was defined as severe EA (2). Delayed sedation is considered to occur if SAS score ≤ 3 upon arrival in PACU. When EA was identified, intermittent intravenous injection of propofol 0.5 mg/kg was performed until the symptoms of agitation disappeared.

We assessed the grade of cough during extubation based on a four-point scale (0 = no cough; 1 = single cough, 2 = persistent cough lasting < 5 s; and 3 = persistent cough lasting ≥ 5 s or bucking). The length of the period following PACU admission to spontaneous breathing, verbal response, and extubation were recorded. The respiratory rate at the time of extubation was also measured. We recorded the hemodynamic parameters including heart rate (HR) and mean arterial pressure (MAP) before anesthesia induction, immediately after intubation, at the end of operation, immediately after extubation, 5 min after extubation, and before leaving the PACU. Desaturation (SpO_2_ < 95%), laryngospasm and other complications (bradycardia, tachycardia, hypotension, and hypertension) were also recorded during the operation and emergence.

In the PACU, score on an 11-point numerical rating scale (NRS) for pain (0 = no pain and 10 = worst pain imaginable), and score on a four-point nausea and vomiting scale (0 = no nausea, 1 = mild nausea, 2 = severe nausea requiring antiemetics, and 3 = retching, vomiting, or both) were evaluated after extubation. Patients were given an injection of sufentanil (5 ug) when NRS was ≥ 4.

### Outcomes

The primary outcome was the incidence of EA defined as the highest SAS score ≥ 5 during emergence. The secondary outcomes were the hemodynamic (HR and MAP) changes at different time points. We also analyzed the operation details (duration of surgery and anesthesia, amount of intraoperative fluid, amount of sufentanil and remifentanil, and intraoperative blood loss), recovery characteristics, and adverse events during the operation and emergence.

### Statistical analysis

Statistical analyses were performed using the software SPSS (ver. 26.0, SPSS, Inc., Chicago, IL, USA). The normality of distribution was assessed based on the Shapiro–Wilk test. According to the normality of the data, the continuous variables were compared by the Student’s *t*- or Mann–Whitney *U* tests, and the categorical variables were evaluated using the χ^2^ or Fisher’s exact test. Repeat-measure variables (HR and MAP) were analyzed using repeated measures ANOVA with Bonferroni correction. A *P*-value < 0.05 was considered statistically significant. All values were expressed as mean (SD), median (range), or number (%).

## Results

**FIGURE 1:**
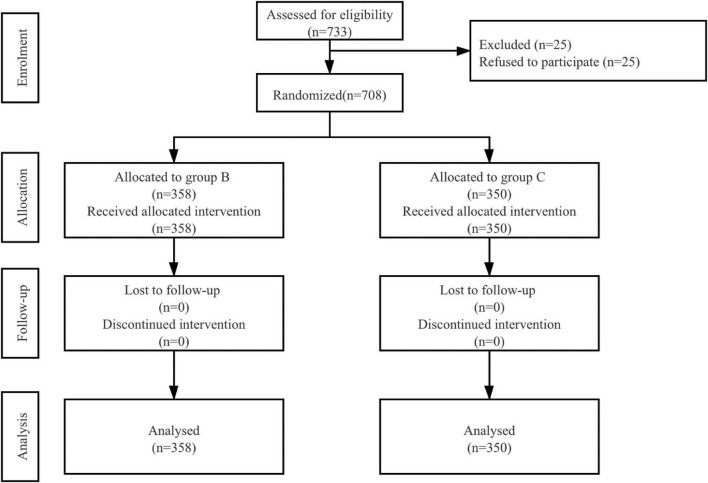
Patient assignment to study group (randomized) and treatment protocols. B, butorphanol; C, control with normal saline.

A total of 733 patients were eligible to be enrolled in the study. Of these, 25 patients refused to participate. In total, 708 patients were randomized and they all completed the study (Figure 1). Patient characteristics and operation details were similar between the two groups (Table 1).

**TABLE 1:** Patient characteristics and operation details.

	Group B (*n* = 358)	Group C (*n* = 350)	*P*-value
Age (yr)	51 (18–65)	51 (18–65)	0.937
Gender (M/F)	223 (135)	211 (139)	0.584
Height (cm)	167 (8)	167 (8)	0.855
Weight (kg)	66 (10)	66 (11)	0.287
BMI	23.62 (2.73)	23.35 (2.69)	0.209
ASA (I/II)	245 (113)	240 (110)	0.969
**Comorbidities**			
Hypertension	61 (17.0%)	49 (14.0%)	0.264
Diabetes	15 (4.2%)	19 (5.4%)	0.441
Heart diseases	32 (8.9%)	23 (6.6%)	0.239
Duration of anesthesia (min)	80 (37)	84 (39)	0.226
Duration of surgery (min)	60 (35)	63 (37)	0.288
Amount of intraoperative fluid (ml)	863 (231)	876 (236)	0.384
Crystal fluid	749 (90)	755 (99)	0.422
Colloidal fluid	113 (209)	120 (214)	0.666
Grade of blood loss	1 (1–3)	1 (1–3)	0.572
Amount of sufentanil (ug)	21 (3)	21 (3)	0.122
Amount of remifentanil (ug)	663 (395)	702 (522)	0.324

Values are mean (SD), median (range), or number (%). B, butorphanol; C, control with normal saline. Grade of blood loss: 1, blood loss ≤ 100 ml; 2, blood loss ≤ 200 ml; 3, blood loss > 200 ml.

**FIGURE 2:**
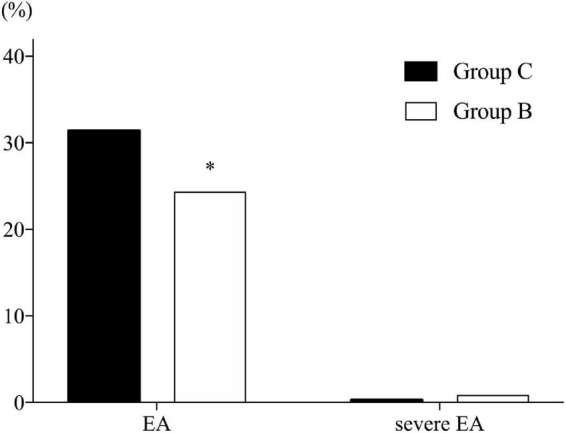
Incidence of emergence agitation. B, butorphanol; C, control with normal saline; EA, emergence agitation. Emergence is defined as the time spent in post-anesthesia care unit (PACU). Agitation is defined as a sedation–agitation scale score ≥ 5. Severe agitation is defined as a sedation–agitation scale score > 5. *P = 0.034 compared with Group C.

The incidence of EA was significantly lower in Group B than in Group C (24.3% vs. 31.4%, respectively; *P* = 0.034). Three patients in Group B and one patient in Group C exhibited severe EA (0.8% vs. 0.3%, respectively; *P* = 0.632), while there was no significant difference (Figure 2).

**FIGURE 3:**
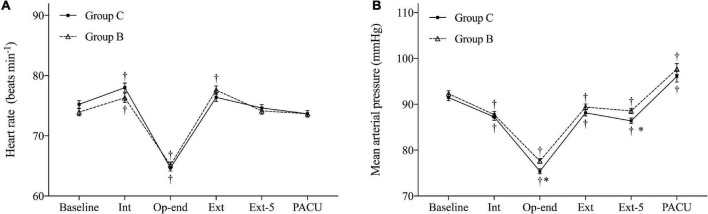
Hemodynamic changes during the operation and emergence. **(A)** Heart rate (HR) and **(B)** mean arterial pressure (MAP). Baseline, before anesthesia induction; Int, intubation; Op-end, end of operation; Ext, extubation; Ext-5, 5 min after extubation; PACU, before leaving PACU. Data are expressed as mean (SD). *P < 0.05 compared with Group C (Bonferroni corrected). ^†^*P* < 0.05 compared with baseline in each group (Bonferroni corrected).

Heart rate (HR) and mean arterial pressure (MAP) during operation and emergence are shown in Figure 3. HR and MAP were similar in both groups at baseline. Furthermore, no HR difference was observed between the two groups. However, the MAP in Group B demonstrated more stable hemodynamic changes at the end of surgery (*P* = 0.004, Bonferroni corrected) and at 5 min after extubation (*P* = 0.008, Bonferroni corrected) compared with Group C.

Parameters related to emergence in the PACU are listed in Table 2. The time from entering PACU to spontaneous breathing (26.5 min vs. 23.7 min, *P* = 0.011), verbal response (36.0 min vs. 33.4 min, *P* = 0.012), and extubation (31.0 min vs. 28.7 min, *P* = 0.025) was longer in Group B compared with Group C. No differences was observed neither in the groups in respiratory rate at extubation nor in the grade of nausea, while the grade of cough was lower in Group B compared with Group C (*P* = 0.024). Moreover, the two groups yielded similar pain scores despite the administration of analgesics and the length of PACU stay.

**TABLE 2:** Recovery characteristics.

	Group B (*n* = 358)	Group C (*n* = 350)	*P*-value
Time to spontaneous breathing (min)	26.5 (13.7)	23.7 (13.0)	0.011
Time to verbal response (min)*	36.0 (14.5)	33.4 (13.9)	0.012
Time to extubation (min)	31.0 (13.5)	28.7 (12.7)	0.025
Respiratory rate at extubation (min^1^)	16 (3)	16 (3)	0.373
Grade of cough at extubation	0.33 (0.58)	0.43 (0.66)	0.024
Grade of nausea after extubation	0 (0–3)	0 (0–3)	0.685
Residual sedation in PACU	56 (15.6%)	43 (12.3%)	0.148
NRS for pain in PACU	1 (0–4)/1.5 (0.7)	1 (0–4)/1.6 (0.9)	0.434
Analgesics in PACU	13 (3.6%)	21 (6.0%)	0.141
Length of PACU stay (min)	50.2 (13.3)	49.2 (12.6)	0.262

Values are mean (SD), median (range), or number (per cent). B, butorphanol; C, control with normal saline; NRS, numerical rating scale; PACU, post-anesthetic care unit. *Verbal response means the participants could answer to the questions in words and to the point.

There was no difference between the groups regarding the incidence of hypertension, hypotension, tachycardia, and bradycardia during the operation. During the PACU, there were fewer patients with hypotension in Group B (0.6% vs. 2.6%, *P* = 0.030) (Table 3). No patients experienced respiratory depression, severe desaturation (SpO_2_ < 90%), and laryngospasm during emergence.

**TABLE 3:** Adverse events.

	Group B (*n* = 358)	Group C (*n* = 350)	*P*-value
**Intraoperative complications**			
Hypertension	33 (9.2%)	26 (7.4%)	0.389
Hypotension	50 (14.0%)	55 (15.7%)	0.513
Tachycardia	7 (2.0%)	15 (4.3%)	0.074
Bradycardia	136 (38.0%)	130 (37.1%)	0.816
**PACU complications**			
Desaturation (SpO_2_ < 95%)	5 (1.4%)	6 (1.7%)	0.733
Hypertension	24 (6.7%)	25 (7.1%)	0.818
Hypotension	2 (0.6%)	9 (2.6%)	0.030
Tachycardia	19 (5.3%)	17 (4.9%)	0.785
Bradycardia	34 (9.5%)	25 (7.1%)	0.257

Values are number (percent). B, butorphanol; C, control with normal saline.

## Discussion

This prospective, double-blinded, randomized study indicated that pre-operative intravenous infusion of butorphanol was effective in reducing the incidence of EA after FESS and making hemodynamics relatively more stable without extending the length of PACU stay.

Prior studies reported that the incidence of EA in adult patients after general anesthesia can reach 20% (9, 20). Male gender, type of surgery, inhalation anesthetics, post-operative pain, and the presence of tracheal and/or urinary catheters are known risk factors for EA (2–5, 9). The incidence of EA after ENT surgery is even higher, almost up to 55.4% (20). Owing to the obstruction of the habitual respiratory channels caused by gauze filling in the nasal cavity to stop bleeding after FESS, awake extubation is preferred after general anesthesia (21). However, awake extubation can lead to a higher agitation incidence. Post-operative pain, as well as the suffocation caused by the gauze strips and blood clots, may be the possible reasons for the high incidence of EA after FESS (22).

The harm of EA is tremendous. It can increase the probability of respiratory and circulatory complications and internal bleeding owing to the excitement of sympathetic nerve, although some patients can relieve themselves. In severe cases, the surgical incision may be ruptured, and the intravenous access and drainage tube may fall off suddenly, leading to the failure of the operation (9). At the same time, the occurrence of EA increases the burden of the medical staff and reduces the satisfaction of patients with disease treatment. At present, analgesic and sedative drugs (such as fentanyl, tramadol, propofol, etc.) are commonly used to prevent and treat EA clinically, but there is a risk of respiratory inhibition or delayed recovery (23–25). Butorphanol is a mixed opioid receptor agonist–antagonist. Its metabolites can act on κ-receptors and have dual effects of activation and antagonism on μ-receptors. It mainly interacts with these receptors in the central nervous system to indirectly exert analgesic, sedative, and other pharmacological effects. Patients have no discomfort, such as agitation and anxiety. Butorphanol usually exerts its effects after intravenous injection within a timeframe of 3–5 min. Its elimination half-life is 2.5–3.5 h, and its analgesic potency is 5–8 times higher than that of morphine (26–28). However, respiratory inhibition rarely occurs, and the incidence of adverse reactions is significantly lower than that of morphine and fentanyl. Based on these characteristics, it may become an ideal drug for post-operative reduction of EA.

Some studies found that among the many causes of EA, post-operative pain may be the most important reason for inducing and aggravating agitation during emergence (20). Butorphanol attracted our attention in the prevention and treatment of EA, owing to the fact that it induces sedation and analgesia without respiratory depression. In this study, we demonstrated that administration of butorphanol before anesthesia induction can effectively reduce the incidence of EA after FESS. We believed that the analgesic and sedative effects of butorphanol are the main reasons for reducing the incidence of EA. In our research, the operation duration was approximately 2–3 h. Therefore, the analgesic and sedative effects of butorphanol were still working on during the emergence duration. This made patients more tolerant to the sense of asphyxia caused by the tracheal catheters and habitual airway blockage. However, in our study, the incidence of EA in the control group was 31.4%, lower than that in previously reported studies (4, 10). This may be attributed to the fact that we gave patients an adequate amount of analgesics during the perioperative period to manage the perioperative pain more efficiently. This could be reflected by the Numerical Rating Scale (NRS) score. In our study, both the highest and mean NRS scores in the control group were lower than those in previously reported results (4, 10). Research studies concerning butorphanol in combination with other drugs used to reduce the incidence of EA are also in progress. Lin et al. (29) found that butorphanol and ketamine combination was more effective than butorphanol or ketamine alone on post-operative EA in patients with gastric cancer. The time to spontaneous breathing, verbal response, and extubation was longer in Group B than that of Group C, while the residual sedation and length of PACU stay yielded no significant differences. Compared with Group C, the grade of cough at extubation in Group B was lower. These results may be attributed to the sedative effect of butorphanol. This medication induced patients in a more appropriate state of sedation, and their recoveries were better and without PACU duration prolongations. All patients were discharged from the PACU when their Aldrete score was ≥ 9. None of the patients experienced drowsiness in the PACU. We performed post-operative follow-up a day after the surgery, and no patients complained of drowsiness. Butorphanol’s sedation effect is dose related. Standard single doses at l–2 mg of butorphanol would cause drowsiness variably at a rate of 0–10% (30). The usage of butorphanol in our research was 20 ug/kg, which was safe for patients. Intravenous use of butorphanol causes analgesia at a fast rate as expected with an onset of l–2 min and maximum relief in 5–30 min. In our research butorphanol was used 1 min before the induction of anesthesia, and the duration of anesthesia was about 80 min. Patients left PACU to the ward 130 min after butorphanol’s administration, which exceeded the peak time of drug action. So it was reasonable that none of the patients experienced drowsiness in PACU or ward.

While HR was similar in both groups during operation and emergence, the MAP at the end of the operation and at 5 min after extubation were significantly higher in Group B. The incidence of hypotension during PACU in Group B was significantly lower compared with that of Group C. These results indicated that the hemodynamics of patients who received a pre-operative intravenous injection of butorphanol were more stable during the perioperative period.

There are several limitations associated with this study. First, we only studied the effectiveness of butorphanol on the incidence of EA in patients aged 18–65 who underwent FESS. Additional studies are warranted for other types of surgery and for children or patients over 65 years. Second, in this study, only one experimental drug dose was set, so, we do not know whether a lower or higher dose of butorphanol can reduce the incidence of EA as well.

## Conclusion

Pre-operative butorphanol infusion decreased the incidence of EA for adult patients who underwent FESS and provided smooth and hemodynamically stable emergence without a concomitant prolongation in PACU stay.

## Statement

Our study adheres to CONSORT guidelines.

## Data availability statement

The original contributions presented in this study are included in the article/supplementary material, further inquiries can be directed to the corresponding authors.

## Ethics statement

The studies involving human participants were reviewed and approved by Renji Hospital Ethics Committee (2017-159). The patients/participants provided their written informed consent to participate in this study.

## Author contributions

XZ, SQ, YZ, JT, DS, and XH: study design. SQ, XZ, YZ, WD, WT, and XH: study conduct. SQ, DS, XH, and ZL: data analysis. SQ, DS, XH, XZ, YZ, and ZL: manuscript preparation. XZ, SQ, ZL, WD, WT, and YZ: data interpretation. All authors contributed to the article, manuscript revision, read, and approved the manuscript.
